# Intramuscular Injection of rAAV2-retro for Low Motor Neuron Transduction: Evaluating Five Promoters

**DOI:** 10.7150/ijms.101807

**Published:** 2025-01-21

**Authors:** Xueqi Gong, Haitong Gao, Wenyuan Wang, Tonghui Xu

**Affiliations:** 1 Laboratory Animal Center, Fudan University, Shanghai 200032, China.; 2 Laboratory Animal Resource Center, Fudan University, Shanghai 200032, China.; 3 Interdisciplinary Research Center on Biology and Chemistry, Shanghai Institute of Organic Chemistry, Chinese academy of Science, Shanghai 200032, China.; 4 Department of Rehabilitation Medicine, Huashan Hospital, Fudan University, Shanghai, China.

**Keywords:** rAAV2-retro, promoter, light-sheet, gene therapy, motor neuron

## Abstract

Recombinant adeno-associated viral vectors (rAAVs) can effectively deliver transgene to the nervous system. The selection of AAV serotype and promoter significantly influences the dynamics of the transgene expression, including its strength and cell-specificity. Previous studies demonstrated that in neonatal mice, the intramuscular (IM) injection of the rAAV2-retro vector could efficiently deliver transgene to lower motor neurons (LMNs) of the brainstem and spinal cord. However, the best promoter for the expression of transgene in the central neural system (CNS) using rAAV2-retro remains undetermined. This study compared five commonly used promoters, including mouse phosphoglycerate kinase (mPGK), CMV early enhancer/chicken β-actin/short β-globulin intron (CAG), human cytomegalovirus (hCMV), chicken β-actin (CBA), and human synapsin (hSyn) promoters. The IM (unilateral gastrocnemius muscle) injection of rAAV2-retro vectors packaged with the reporter constructs containing each promoter was performed in the newborn C57BL/6J mice. The levels of gene expression and the types of cells were examined using the light-sheet illumination imaging technique and confocal microscopy. Our findings revealed that rAAV2-retro primarily targeted the brainstem and spinal cord within the CNS. Among the five promoters tested, CAG and hCMV showed the highest gene expression. Almost all the transduced cells were identified as LMNs. Additionally, gene expression driven by hCMV was found to be dependent of the inclusion of WPRE and β-globin intron elements. Importantly, none of the promoters induced hepatotoxicity, ensuring the safety of rAAV2-retro-mediated expression. This study provided valuable insights for optimizing the rAAV2-retro-mediated gene delivery system to LMNs in the brainstem and spinal cord, which might have potential implications for research on motor neuron-related diseases.

## Introduction

Studies have identified numerous evolutionarily diverse recombinant adeno-associated virus (rAAV) and their wide applications in gene therapy. Clinical and preclinical studies have demonstrated various advantages of these vectors, such as sustained long-term expression of foreign gene, lack of pathogenicity in animal models, and their ability to infect different tissues 1-3. In particular, some of them can efficiently transduce the central nervous system (CNS) neurons. rAAV vectors are favored as viral vectors for targeting CNS neurons due to their low immunogenicity and a mechanism primarily based on the formation of episomal DNA in the transduced cells nuclei to initiate transgene expression 4-6. They have already been employed in clinical trials in neurology 7-9.

rAAV2-retro has emerged as a robust vector, demonstrating a substantial enhancement in retrogradely transducing projection neurons within the brain compared to commonly used AAV serotypes 10. This vector has significantly facilitated the functional dissection of mammalian central neural circuits. Moreover, the rAAV2-retro-based tool has been successfully employed to therapeutically intervene the diseases, which are characterized by the progressive dysfunction of network. For instance, Z. Lu's lab recently reported the development and application of a retrograde rAAV2-retro-based strategy as a therapeutic approach to isolate and modulate D1-MSNs precisely, leading to the rescue of motor defects in Parkinson's disease models (both primate and mouse models) 11. Our recent study showed that a one-time rAAV2-retro injection into a single muscle efficiently and extensively transduced the brainstem and spinal cord lower motor neurons (LMNs) 12, suggesting therapeutic potential for motor neuron diseases (MNDs).

MNDs encompass numerous progressive neurological diseases resulting from dysfunction in either upper motor neurons in the frontal lobe or lower motor neurons in the spinal cord and brainstem. These diseases are characterized by symptoms, including muscle weakness, respiratory insufficiency, and loss of ambulation, often leading to early death. Among MNDs, SMA (spinal muscular atrophy) and ALS (amyotrophic lateral sclerosis) have the highest prevalence and are the severe ones. Current studies have proved gene therapy as a promising strategy to treat MNDs, with motor neurons being the primary target. However, the challenge lies in specifically and efficiently transducing motor neurons, particularly LMNs distributed throughout the spinal cord and brainstem. The intramuscular injection (IM) can extensively and efficiently achieve rAAV2-retro transduction of LMNs; therefore, the IM of rAAV2-retro presents a potential strategy for MND gene therapy. Delivering the AAV through IM injection offers a non-invasive, quick, easy, and safe administration route, which is feasible in various settings.

Moreover, AAV vectors, following IM delivery, have demonstrated long-term as well as stable expression of transgene in various animal models and clinical trials 13-15. However, a limitation of IM delivery is the restricted transduction to cells around the injection site, with relatively low efficiency in the CNS, even with large-volume injections. In gene therapy and functional studies, enhancing the AAV vector-mediated expression of a transgene holds the potential for better therapeutic outcomes. Therefore, optimizing the expression of LMNs by rAAV2-retro following IM delivery is crucial for advancing preclinical and clinical therapies for MNDs.

In addition to the AAV capsid and administration method, the choice of promoter is also crucial for determining the efficiency of transduction. In the genome of viral vectors, promoter is a key DNA element, which regulates the cell-specific expression of transgene as well as the strength of expression. The cellular transcription factors are responsible for its activation. Therefore, selecting an optimal promoter is crucial for enhancing, stabilizing gene expression and designing AAV vector 16. Consequently, promoter selection is a crucial aspect of AAV vector design. To date, an AAV with a specific promoter that induces robust and selective motor neuron-specific expression of transgene has not been identified through direct comparison.

The current study aimed to evaluate the potential of different promoters in the rAAV2-retro-mediated gene transfer to LMNs in the spinal cord and brainstem, contributing to the development of promising therapeutic approaches for MNDs.

## Materials and Methods

### AAVs

In this study, Brain VTA (Brain VTA Co., Ltd., Wuhan, PR China) packaged and provided all the AAVs. The standard plasmid transfection protocols were used to package the plasmids into AAV vector serotype 2-retro. The human embryonic kidney 293T (HEK293T) cells were used to extract AAVs. Iodixanol gradient ultracentrifugation was used to purify the AAV particles after the HEK293 cell lysis. qPCR was used to determine the viral titers.

### Animals and intramuscular injection

The mice (C57BL/6 mice) used in this study were obtained from SLAC ANIMAL (Shanghai, China). All the experiments were conducted under the guidelines of the care and use of laboratory animals of Fudan University (Shanghai, China) and approved by the Animal Advisory Committee at Fudan University. A total of 51 mice were used in this study, with both male and female mice randomly assigned to the experimental groups to ensure gender diversity. Experimental animals were housed in SPF-grade facilities with controlled temperature, humidity, lighting, and noise levels. A 12-hour light/dark cycle was maintained, and mice had ad libitum access to food and water. Pregnant females were monitored daily, and neonatal mice were undisturbed for three days postpartum. After the injection, the mice were meticulously handled to guarantee their recovery.

Cold aluminum plates were used to induce hypothermic anesthesia in P4 mouse, as described previously 12. For the IM injection, ophthalmic scissors were used to make an incision in the right hindlimb of the pup in order to expose the gastrocnemius muscle. All viruses were diluted with phosphate buffer solution to a concentration of 5 X 10^12^ genome copies /mL, and a total volume of 2μL containing 1 X 10^10^ genome copies of each vector was administered per P4 pup via IM injection. We employed a glass micropipette linked to a high-precision syringe pump (item no. 53311, Quintessential Stereo taxic Injector, Stoelting, Wood Dale, IL, USA) to achieve uniform regulation of the injection speed at 1.0 μL/min. After injection, the micropipette was held for 30 sec and then withdrawn slowly from the muscle.

### Primary neurons culture

Primary neurons were isolated from fetal mouse cerebral cortex on gestation day 16. Cortical tissues were dissociated with papain and plated on PDL-coated 24-well plates. Cells were initially cultured in Neurobasal medium containing 10% FBS, 1X GlutaMAX, and P/S at 37°C for 4 hours. After this period, the medium was replaced with a maintenance medium without FBS but containing 1X B27 and other necessary supplements. All cells were maintained in a humidified incubator.

### Tissue preparation and immunofluorescence

Isoflurane was used to anesthetize the mice, followed by transcardial perfusion with 1×PBS (ice-cold) and fixation with 4% PFA (paraformaldehyde, freshly prepared and ice-cold) in 1× PBS. Brain, spinal cord, liver, and dorsal root ganglion were excised, followed by post-fixation at 4°C for 48 h in 4% PFA. For immunohistochemical (IHC) quantification, the sampling protocol was as follows: Spinal cord sections were obtained from L1-L3 segments, fixed, dehydrated, embedded in paraffin, and cut into 8 μm slices. Every 20th section was stained and analyzed. Brain tissue was sampled from the region approximately between Lateral -2.4 mm and Lateral 2.4 mm, processed similarly, and cut into 8 μm sagittal slices, with every 20th section used for IHC. Liver sections were taken from the main lobe, fixed, dehydrated, embedded, and cut into 3 μm slices, with every 30th section analyzed. At least 4 sections were imaged and analyzed, each corresponding to different subregions. Xylene was used to deparaffinize the slides, which were then rehydrated with gradient ethanol, followed by retrieving antigen using heat-induced epitope retrieval (HIER). For antigen retrieval, all tissue sections were subjected to microwave heat-induced epitope retrieval using Tris-EDTA buffer at pH 9.0 (Solarbio, C1038). Then, the non-specific binding sites and endogenous peroxidase were blocked with 5% normal goat serum, followed by overnight incubation with primary antibodies (16-20 h) at 4℃. The following primary antibodies were used: rabbit anti-Chat (1:1000, Abcam, ab178850) and mouse anti-eGFP (1:1000, Santa Cruz Biotechnology, sc-5384). The tissues were then washed, followed by incubation for 1-2 h with the respective secondary antibodies at room temperature. The secondary antibodies included donkey anti-mouse 488 (1:500, Thermo Fisher, A-20102) and donkey anti-Rabbit 555 (1:500, Thermo Fisher, A-31572). DAPI (1:1,000, Sigma-Aldrich, 10236276001) in PBS was used for staining the nuclei for 10 minutes. The samples were then washed and mounted in an antifade solution (Fluoromount-G, 0100-01, SouthernBiotech).

### Tissue clearing

Mice were perfused with 1×PBS. Their spinal cord was then dissected out, fixed in 4% PFA at 4℃ overnight, and washed with PBS thrice. As described in the original study, the samples were processed using the FDISCO clearing procedure 17. Briefly, this is a two-step process, which includes dehydration and refractive index matching. Then, the tissue samples were treated with the gradient solutions of peroxide-free tetrahydrofuran (THF, 80124428, Sinopharm Chemical Reagent). These gradient solutions (100%, 80%, 70%, and 50% trimethylamine with pH 9.0) were prepared by mixing with distilled H_2_O [dH_2_O] (T103287, Aladdin Reagent, Shanghai, China) at 4℃ on a shaker (1 hour per gradient). In order to perform refractive index matching, Dibenzyl ether (DBE) solution (D807110, Macklin) was used. In order to remove the peroxides in DBE and THF, 100% THF was passed through a chromatography column, which was filled with basic activated aluminum oxide (10000918, Sinopharm Chemical Reagent) 18.

### Microscopy

The images of cultured cortical neurons were captured using a confocal microscope (SP8, Leica) equipped with an X20 objective lens. The specific settings employed were as follows: emitted light at 488 nm, laser intensity set to 5%, gain at 800, and stained with ALEXA488 dye; emitted light at 405 nm, laser intensity set to 2%, gain at 800, and stained with ALEXA488 dye. High-resolution images were generated in Z-stack format consisting of 30 optical sections with a step size of 0.5 μm.

For imaging the tissue slides, a digital slide scanner (PANNORAMIC 250 Flash III, 3DHISTECH) with an X20 objective lens was used. High-magnification images of tissue slides were obtained using the SP8 confocal microscope with an X40 oil-immersion objective for representative imaging. For eGFP, we used settings of 488 nm emitted light, 5% laser intensity, and 800 gain. Choline acetyltransferase (ChAT) was detected with 561 nm emitted light, 5% laser intensity, and 1000 gain. DAPI staining was visualized with 405 nm emitted light, 3% laser intensity, and 800 gain. High-resolution Z-stack images, comprising 10-15 optical sections with step sizes of 0.3 μm for liver slices or 0.5 μm for brain and spinal cord slices and, were captured, ensuring comprehensive visualization of cellular structures.

For imaging spinal cord samples after tissue clearing, a light sheet fluorescence microscope (LS18, Nuohai Life Science) with a 4X objective lens was employed. The parameters used for image acquisition included an excitation light at 488 nm, laser intensity set to 5%, while the gain and step size were set to the default settings of the microscope. Imaris software was used to analyze and reconstruct (3D reconstructions) the obtained images.

For imaging the DRGs which from the L1 segment of the spinal cord, an SP8 confocal microscope with an X40-oil objective was used. The specific settings employed were as follows: emitted light at 488 nm, laser intensity set to 2%, gain at 800, and stained with ALEXA488 dye. High-resolution images were generated in Z-stack format consisting of about 10 optical sections with a step size of 10 μm.

### Quantification of histological samples

Cells were quantified using a combination of QuPath Land Imaris software. TIFF files were imported into QuPath, where regions of interest (ROIs) were annotated. Subsequently, cell nuclei labeled with the DAPI channel within these annotated areas were identified using the 'cell detection' feature, generating detections. These detections were then classified using the 'Classify' function, with parameters adjusted to ensure accurate recognition of all eGFP-labeled cells. Finally, the images were subjected to 3D reconstruction in Imaris, followed by manual verification to ensure accuracy.

To quantify the fluorescence intensity, we utilized ImageJ software for image analysis. The process involved importing the microscopy images into ImageJ, followed by the separation of multi-channel datasets to isolate the fluorescent signals. Regions of interest (ROIs) were then defined based on the observed fluorescence. Subsequently, thresholds were adjusted according to the background intensity to ensure accurate quantification. Finally, we measured the "Area" and "Integrated Intensity" within these ROIs to obtain the quantitative data for fluorescence intensity.

For cellular colocalization analysis, we imported monochromatic TIFF images into ImageJ, separated multi-channel datasets, and identified ROIs based on cellular features. Channels were aligned for precision, and colocalization was visualized using heat maps and scatterplots. Quantification was performed using Pearson's and Manders' coefficients, and the results were exported for rigorous statistical analysis to ensure accurate colocalization assessment.

### Statistical analyses

All the values were presented as mean ± SEM (standard error mean). The differences between two groups and among more than two groups were analyzed using Student's *t*-tests and one-way ANOVA followed by Tukey's post-hoc test, respectively. Statistical significance was defined at P <0.05 (*P <0.05, **P <0.01, and ***P <0.001, ****P <0.0001).

## Results

### Procedure for assessing rAAV2-retro-mediated transgene expression levels driven by diverse promoters

**Figure 1:**
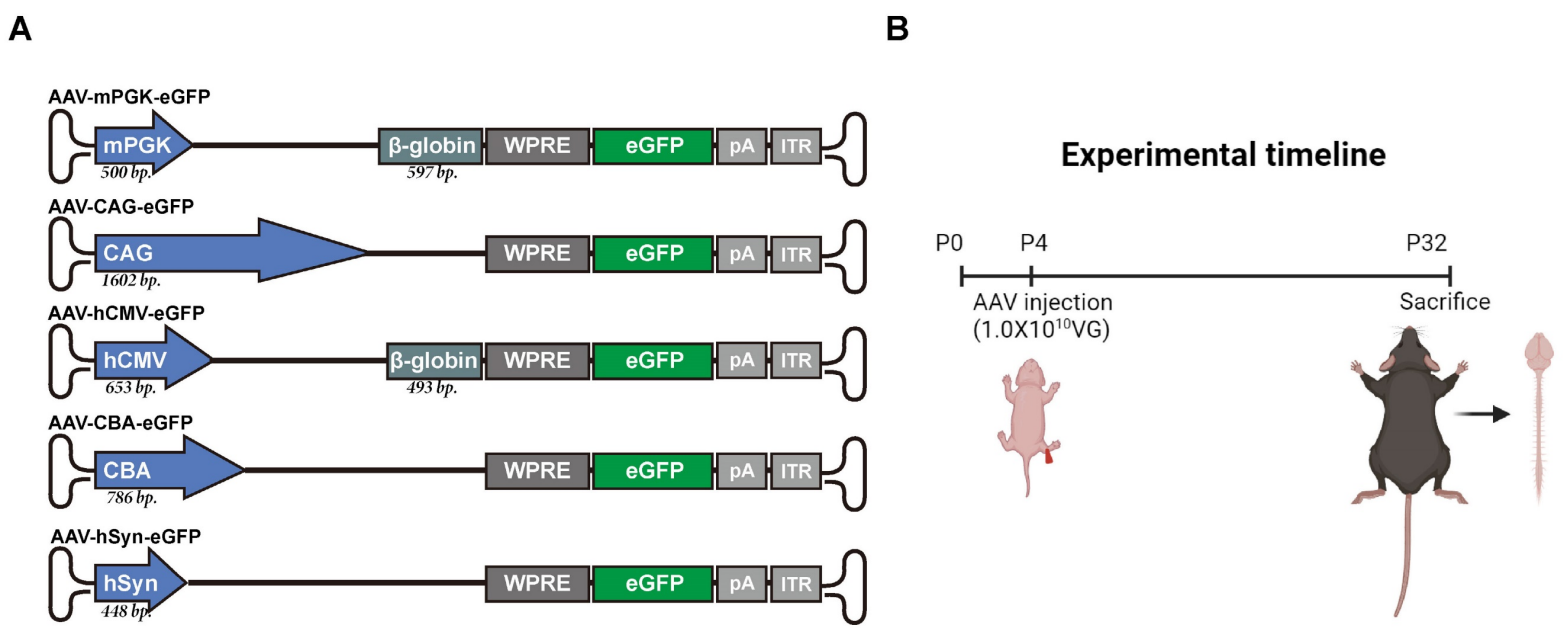
** Schematic representation of the study design.** (A) Five vectors expressing an eGFP (720 bp) activated by different promoters. The mPGK promoter (500 bp) is upstream of a β-globin intron (597 bp). CAG promoter is 1602 bp long. The human CMV (hCMV) promoter (653 bp) is upstream of a β-globin intron (493 bp). CBA promoter is 786 bp long. The human SYN (hSYN) is the shortest promoter with a 448-bp size. Inverted terminal repeats (ITR) flank the packaging cassettes. Bp: base pairs, pA: polyadenylation signal. (B) Illustration of the vectors induction experimental workflow. rAAV2-retro with different promoters was injected in the right gastrocnemius of the P4 newborn C57BL/6J mice. The mice were killed after four weeks, and the expression profiles at cell level as well as expression levels of transgene were identified in the CNS.

This study compared the expression levels of transgene under five different rAAV2-retro-deliverd promoters in newborn mice. These promoters included mouse phosphoglycerate kinase (mPGK), CMV early enhancer/chicken β-actin/short β-globulin intron (CAG), human cytomegalovirus (hCMV), chicken β-actin (CBA), and human synapsin (hSyn) (Figure 1A). The selection of these promoters was depended on their use in transducing the nervous system and their relatively small size, which allowed them to accommodate large transgenes. Fluorescent reporter-enhanced green fluorescent protein (eGFP) was tagged to each promoter in order to assess transgene expression strength. Reporter constructed with different promoters were encapsulated in rAAV2-retro and injected into the unilateral gastrocnemius of newborn C57BL/6J mice on postnatal day 4. The levels of expression in the spinal cord and brain were assessed 4 weeks (a commonly used time point for gene therapy in rodents 19) following IM delivery (Figure 1B).

### Assessing the ability of gene expression from the AAV vectors in cortical neurons *in vitro*

**Figure 2:**
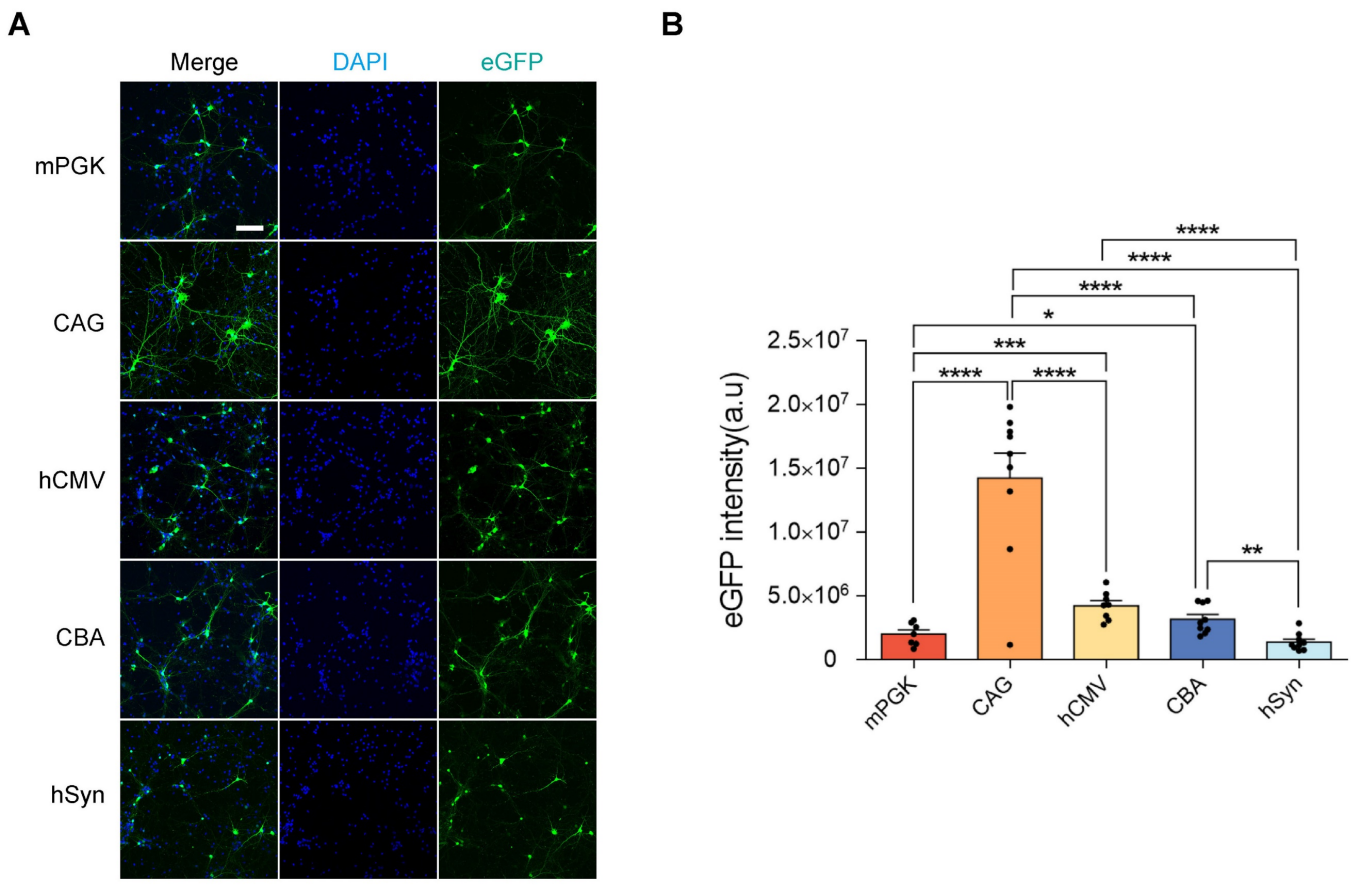
** eGFP expression level comparison in rAAV2-retro vector-transfected cortical neurons under different promoters.** (A) rAAV2-retro vector-transfected 14 DIV cortical neurons: mPGK-eGFP, CAG-eGFP, hCMV-eGFP, CBA-eGFP, and hSyn-eGFP (scale bar, 100μm). (B) Mean intrinsic eGFP intensity quantification in cortical neurons. F (4, 37) = 29.43, P < 0.0001. Data presented as mean ± SEM (mPGK n=7; CAG n=9; hCMV n=8; CBA n=9; hSyn n=9). *P <0.05, **P <0.01, ***P <0.001, ****P <0.0001, one-way ANOVA with Tukey's multiple comparison test.

The rAAV2-retro vectors, containing different promoters, were transfected into E16 mice cortical neurons to assess their functionality* in vitro*. Following transfection, all five vectors induced eGFP expression, confirming the efficacy of the rAAV2-retro vectors in cortical neurons (Figure 2A). To determine the strength of gene expression levels driven by different promoters *in vitro*, the eGFP intensity was measured. After 48 hours, the intensity of eGFP was comparable between different promoters, and the CAG exhibited the highest intensity, while the mPGK and SYN showed the lowest intensities (Figure 2B). However, it is crucial to point out that the transfection of cell lines *in vitro* does not accurately represent AAV transduction *in vivo*. Therefore, the rAAV2-retro vectors carrying different promoters were directly compared (side-by-side) to evaluate their transgene expression driving ability in the CNS *in vivo*.

### *In vivo* efficiency of the five promoters in transducing brain

**Figure 3:**
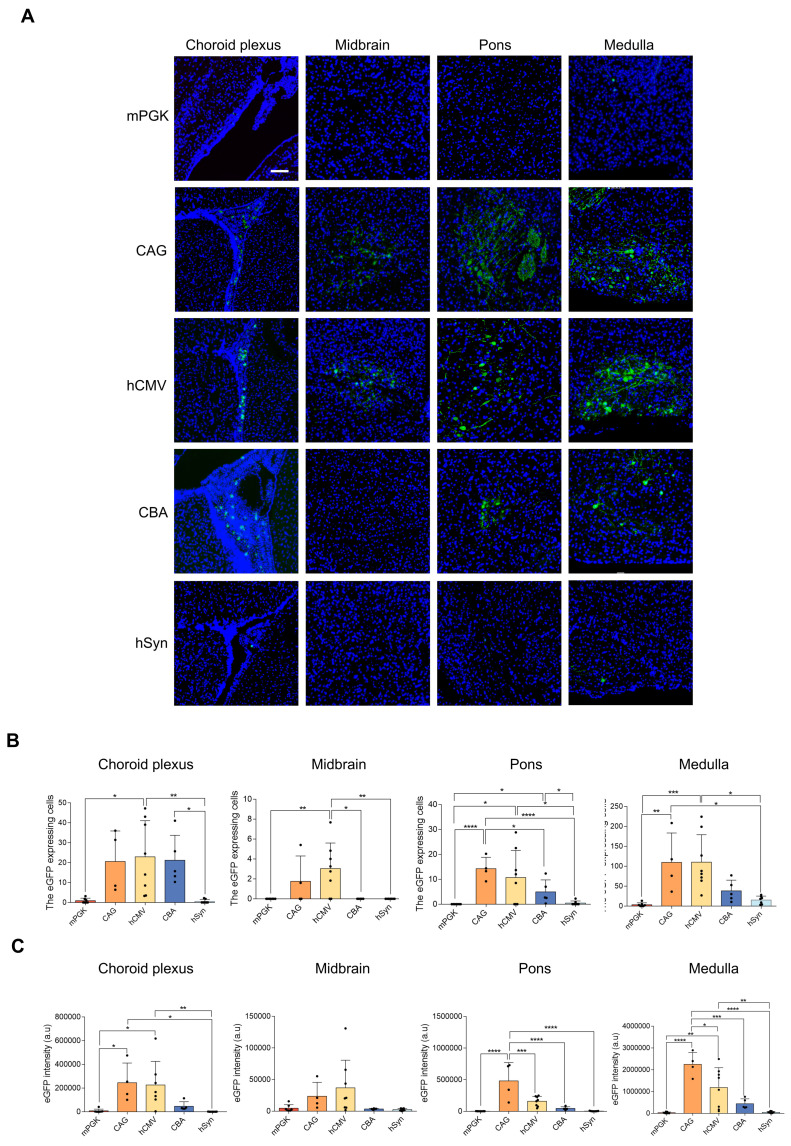
** eGFP expression level comparison in rAAV2-retro vector-transfected brain under different promoters.** (A) Different subregions of the brain confocal imaging for P4 newborn C57BL/6J mice injected with rAAV2-retro containing different promoters into the right gastrocnemius (scale bar, 100μm). The 8-μm-thick brain slices were stained for eGFP (green) and DAPI (blue). (B) Numbers of native eGFP-expressing cells in different subregions of the brain. Choroid plexus: F (4, 26) = 6.125, P = 0.0013; Midbrain: F (4, 26) = 5.453, P = 0.0025; Pons: F (4, 26) = 6.040, P = 0.0014; Medulla: F (4, 26) = 8.266, P = 0.0002. One-way ANOVA with Tukey's multiple comparison test. (C) eGFP fluorescence intensity of different subregions of the brain. Choroid plexus: F (4, 25) = 6.432, P = 0.0011; Midbrain: F (4, 26) = 2.867, P = 0.0431; Pons: F (4, 26) = 16.44, P < 0.0001; Medulla: F (4, 26) = 16.98, P < 0.0001. Data presented as mean ± SEM (mPGK n=7; CAG n=4; hCMV n=8; CBA n=5; hSyn n=7). *P <0.05, **P <0.01, ***P <0.001, ****P <0.0001, one-way ANOVA with Tukey's multiple comparison test.

**Figure 4:**
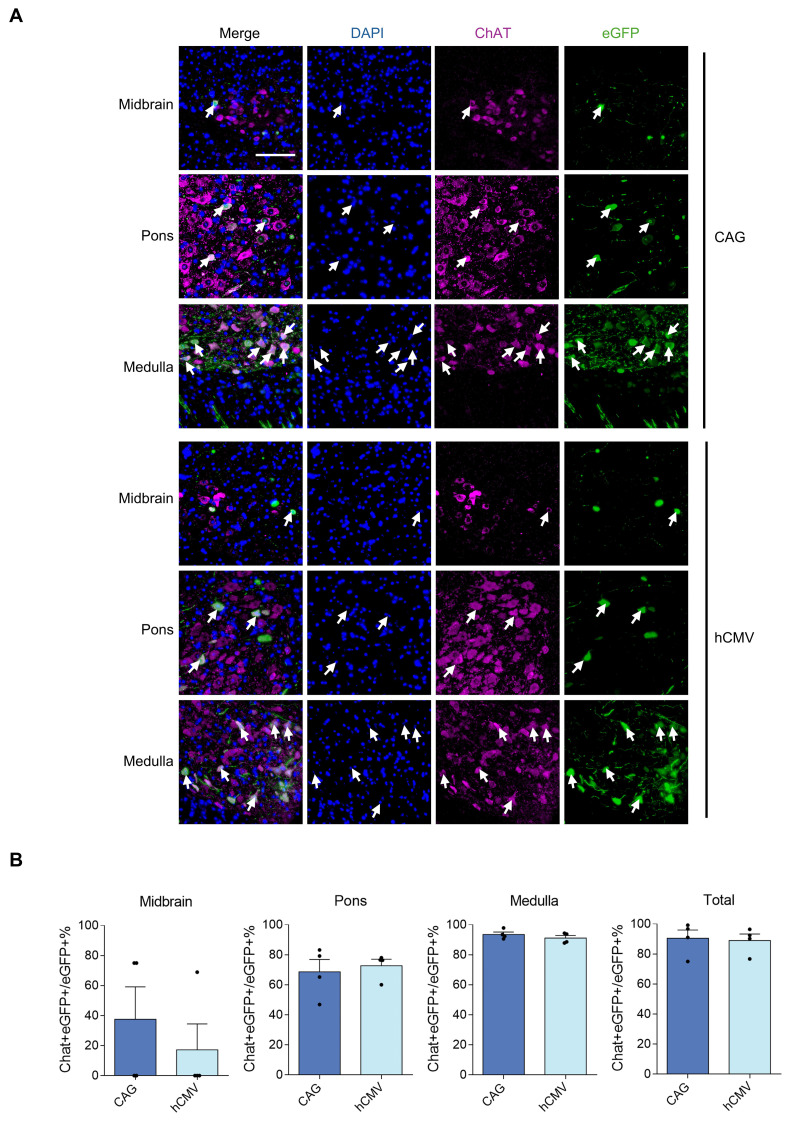
** rAAV2-retro-tranfected cells containing CAG and hCMV in the brainstem stained with ChAT antibody.** (A) Double-labeled brainstem sections illustrating the expression of eGFP in the motor neurons labeled with ChAT antibody. The tailed arrows highlight cholinergic motor neurons. Blue: DAPI, green: eGFP, red: ChAT (scale bars, 100 µm). (B) Percentage of eGFP/ChAT+ motor neurons in the brainstem. Midbrain: F(3,3)=1.577, P=0.7174. Pons: F(3,3)=3.710, P=0.3101; Medulla: F(3,3)=1.187, P=0.8914; Total: F(3,3)=1.600 P=0.7088. Data are shown as means ± SEM (n=4 mice for each group), two-tailed unpaired *t*-test.

All 5 rAAV2-retro vectors were injected into the right gastrocnemius of newborn C57BL/6J mice on postnatal day 4. After four weeks, a careful dissection of the intact brains and spinal cords was performed. Using a digital slide scanner to detect brain slices, the retrograde expression efficiency of rAAV2-retro vectors containing different promoters in the brain following IM delivery was evaluated. The results revealed that the rAAV2-retro vectors primarily transduced the brainstem, including the midbrain, pons, and medulla. In contrast, in the choroid plexus, fewer cells were transduced (Figure 3A). The number of transduced cells, namely the eGFP-positive cells, in these subregions of brain was counted respecively. The results showed that the CAG and hCMV transduced the most cells, which were close to each other (Figure 3B), in each subregion. The CBA also transduced a small number of cells, while the mPGK and hSyn showed minimal expression. We also measured the eGFP fluorescence intensity in these subregions separately and compared it across different promoters. The results exhibited a trend similar to that observed in the number of transduced cells, wherein the CAG and hCMV promoters transduced the highest eGFP intensity, followed by the CBA promoter, while the mPGK and hSyn promoters showed minimal expression. (Figure 3C). In order to ascertain the proportion of LMNs in transduced cells directed by CAG and hCMV in the brainstem, choline acetyltransferase (ChAT), a cholinergic LMN marker, was used. We conducted separate analyses on the three subregions of the brainstem and found that the ratios of cholinergic LMNs among the transduced cells directed by CAG and hCMV promoters were highest in the medulla. In contrast, these ratios were relatively lower in the midbrain and pons. However, due to the smaller number of transduced cells in the midbrain and pons directed by CAG and hCMV promoters, the overall ratios of cholinergic LMNs among the transduced cells directed by these promoters in the brainstem remained high, reaching 90.49±5.44% and 88.55±6.05%, respectively (Figure 4).

### *In vivo* efficiency of the five promoters in transducing spinal cord

**Figure 5:**
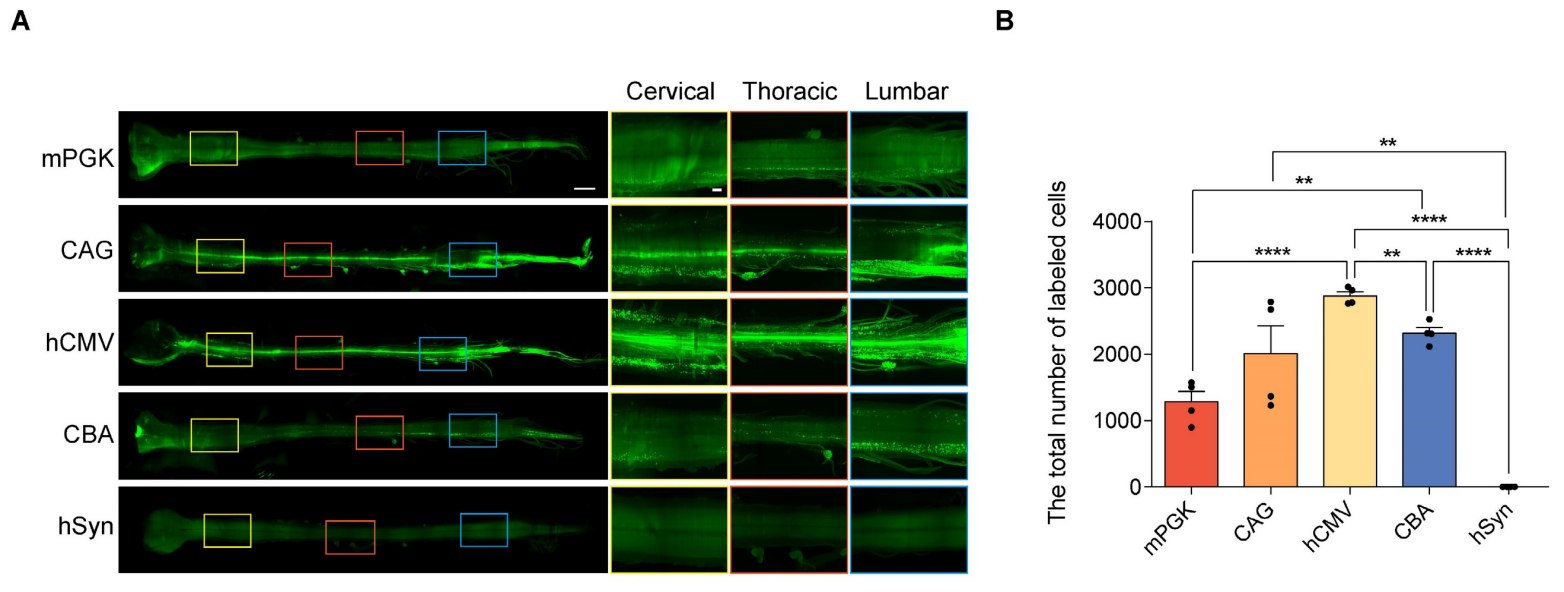
** Comparison of eGFP expression driven by different promoters in the spinal cord transfected with rAAV2-retro vectors.** (A) Spinal cord light-sheet imaging for P4 newborn C57BL/6J mice injected with rAAV2-retro containing different promoters into the right gastrocnemius. The left panels show an overview of the spinal cord (scale bar, 1500 µm). The right panels show magnified images, which were taken at the inset in the left panels, revealing the magnified horizontal views of the cervical, thoracic, and lumbar spinal cord (scale bar, 300 µm). (B) Total number of native eGFP-expressing cells in the spinal cord. F(4,15) = 29.53, P < 0.0001. Data are shown as means ± SEM (n=4 mice for each group). **P <0.01, ****P <0.0001, one-way ANOVA with Tukey's multiple comparison test.

**Figure 6:**
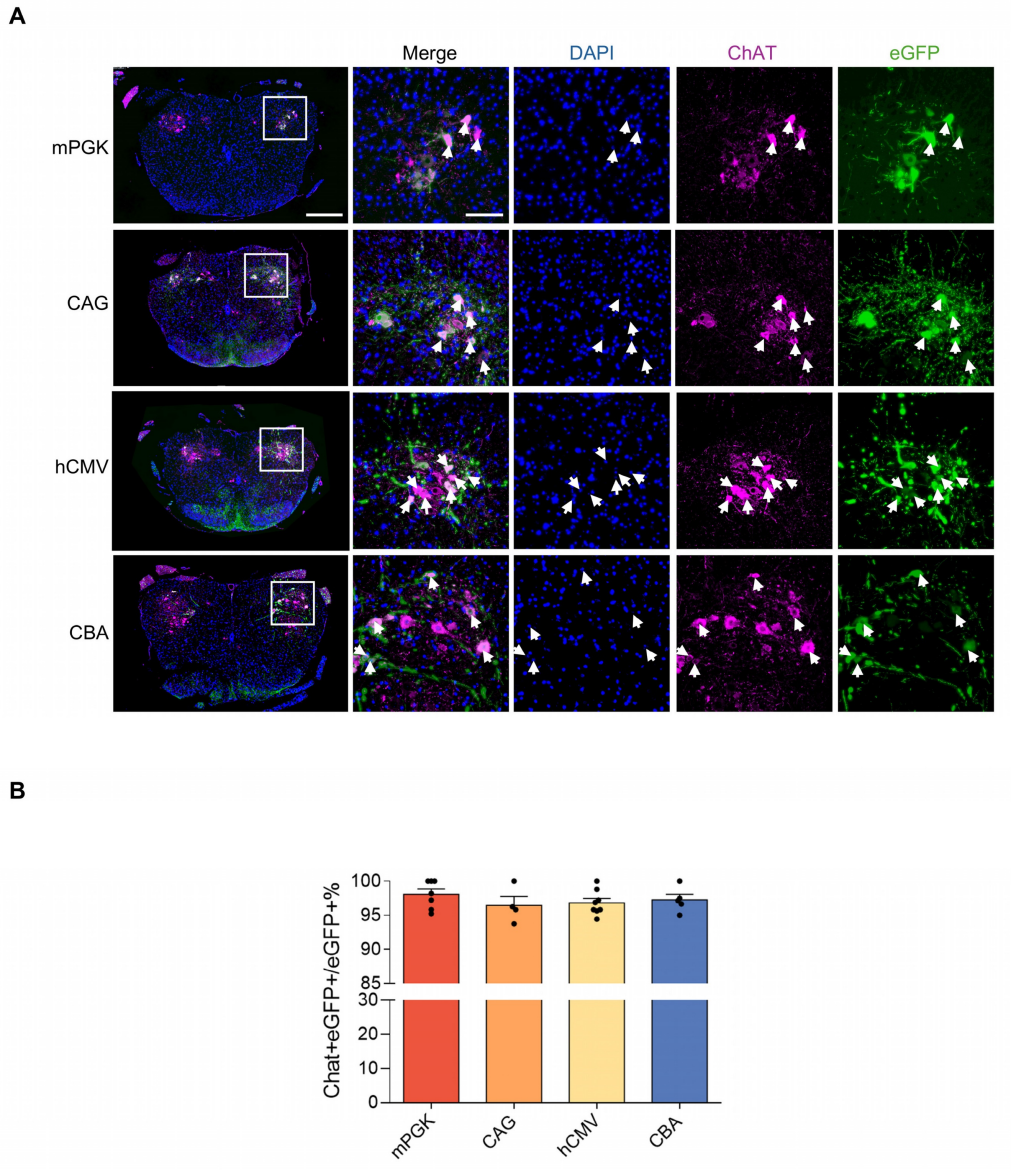
** CAG and hCMV-containing rAAV2-retro-transduced cells in the spinal cord stained with ChAT antibody.** (A) eGFP expression in ChAT-labeled motor neurons in the double labeled spinal cord sections. Blue: DAPI, green: eGFP, red: ChAT. The leftmost panels show the transverse lumbar spinal cord sections (scale bar, 400 µm). The right panels show magnified images, which were taken at the inset in the left panels (scale bar, 100 µm). Arrowheads highlight cholinergic motor neurons. (B) Percentage of eGFP/ChAT+ motor neurons in the spinal cord. F(3, 20) = 0.7029, P = 0.5614. Data are shown as means ± SEM (mPGK n=7; CAG n=4; hCMV n=8; CBA n=5), one-way ANOVA with Tukey's multiple comparison test.

Subsequently, the expression efficiencies of these five promoters were compared within the spinal cord. After intramuscular (IM) delivery, we compared the retrograde expression efficiency of rAAV2-retro vectors harboring various promoters in the anterior horn of the spinal cord—the primary location of lower motor neurons—using tissue clearing, fluorescent imaging, and 3D reconstruction techniques. The findings revealed that except hSyn, all the promoters, including mPKG, CAG, hCMV, and CBA, facilitated noticeable expression in various parts of the spinal cord, including cervical, thoracic, and lumbar regions (Figure 5A). Quantitative assessment showed that the number of transduced cells driven by mPKG, CAG, hCMV, and CBA were 1282±158, 2014±416, 2878±63, and 2317±84, respectively (Figure 5B). Moreover, immunostaining with the ChAT antibody indicated that mPGK, CAG, hCMV, and CBA promoters transduced a high proportion of cholinergic neurons (98.07±0.76%, 96.46±1.30 %, 96.83±0.64 %, and 96.46±1.30 %, respectively; Figure 6A and 6B).

It should be noted that upon closer examination of the transverse spinal cord sections, it became evident that the CMV and CAG promoters not only drive robust eGFP expression in motor neurons but also in the white matter track axons (Supplementary Figure S1). These results underscore the ability of CMV and CAG promoters to facilitate transgene expression in multiple cellular components within the spinal cord following IM of rAAV2-retro vectors.

In addition, following the processes of tissue clearing and light sheet imaging, we observed virtually no hSyn-mediated eGFP-positive neurons within the spinal cord. However, in the IHC experiment, through sectioning the spinal cord and staining with a GFP antibody, we did observe a very small and sparsely distributed population of hSyn-mediated eGFP-positive neurons. The apparent contradiction between these two sets of results is likely attributed to the fact that the clearing procedure tends to quench fluorescent signals, often leading to the originally weak fluorescent signals becoming difficult to detect 17. Additionally, due to the extremely low abundance of hSyn-mediated eGFP-positive neurons, we excluded the hSyn results from our comparison of the percentage of eGFP/ChAT+ motor neurons in the spinal cord.

Furthermore, a substantial cell expression was observed within the dorsal root ganglia (DRGs) following the IM administration of rAAV2-retro vectors containing each promoter (Supplementary Figure S2A). The results revealed that rAAV2-retro vectors carrying CAG, CMV, and CBA promoters achieved approximately 4-8-fold higher expression efficiency of DRGs compared to those with mPGK and hSyn promoters (Supplementary Figure S2B).

### Dependence of efficient expression of rAAV2-retro vectors driven by hCMV on the WPRE and β-globin intron

**Figure 7:**
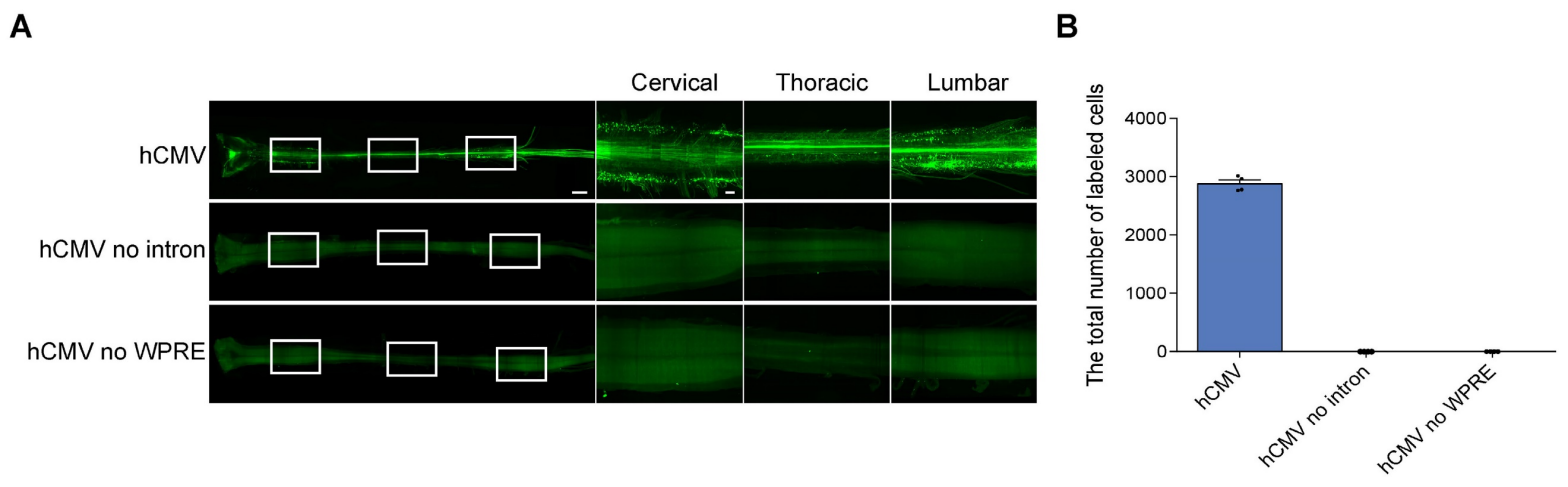
** Effects of intron and WPRE on eGFP expression levels in the spinal cord transfected with rAAV2-retro containing hCMV.**(A) Spinal cord confocal imaging for the P4 newborn C57BL/6J mice injected with rAAV2-retro harboring hCMV expressing eGFP with intron and WPRE, without intron, and without WPRE into the right unilateral gastrocnemius. The left panels illustrate an overview of the spinal cord (scale bar, 500 µm). The right panels show magnified images, which were taken at the inset in the left panels revealing the enlarged horizontal views of the cervical, thoracic, and lumbar spinal cord (scale bars, 300 µm). (B) Total number of native eGFP-expressing cells in the spinal cord with intron and WPRE, without intron, and without WPRE (error bars indicate mean ± SEM; n = 4 mice for each group).

The strategies aimed at improving the expression of transgene using rAAV vectors include the modification of the vector's capsid and incorporation of expression cassettes, such as CMV enhancers, introns, and polyadenylation signals 20. Previous studies showed that adding elements, such as WPRE (woodchuck hepatitis post-transcriptional regulatory element) and β-globin intron, could enhance transgene expression 21-23. In the current study, all rAAV2-retro vectors with different promoters included WPRE and those with hCMV and mPGK also contained β-globin intron. The rAAV2-retro vector with hCMV achieved robust expression in the CNS* in vivo*. Therefore, the dependency of this efficient expression on the WPRE and β-globin intron was further investigated. For this purpose, rAAV2-retro-hCMV-eGFP without WPRE or β-globin intron were compared to the construct rAAV2-retro-hCMV-eGFP containing both elements. Quantifying the eGFP-expressing cells in the spinal cord revealed that the absence of either WPRE or β-globin intron significantly impaired the efficiency of rAAV2-retro vector expression driven by hCMV, lacking eGFP+ cells in the whole spinal cord (Figure 7A and 7B). Therefore, including either WPRE or the β-globin intron in the vector was necessary for the efficient expression by hCMV in the CNS* in vivo*.

### *In vivo* efficiency of the five promoters to transduce liver

**Figure 8:**
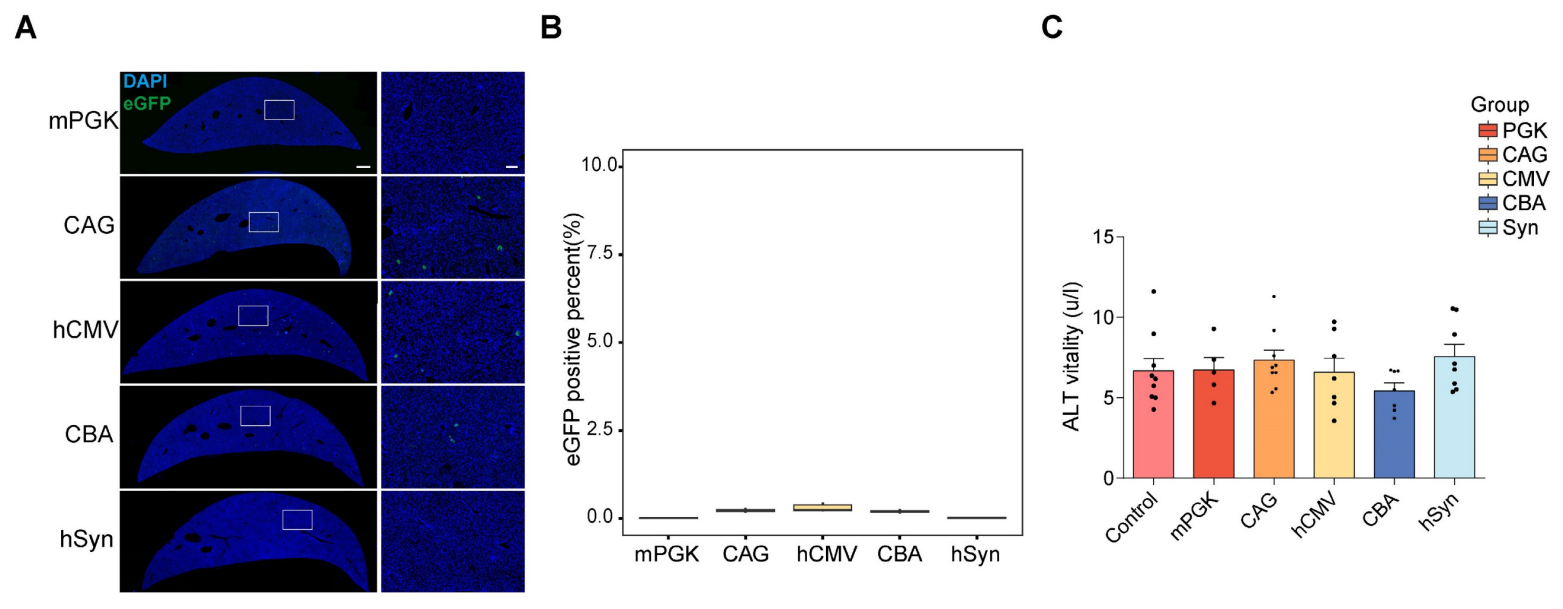
** Liver expression efficiency of the five promoters *in vivo*.** (A) eGFP expression mediated by AAV vector in the liver. The left panels display the representative sections of the liver (scale bar, 500 µm). The right panels show magnified images, which were taken at the inset in the left panels (scale bars, 100 µm). (B) Percentage of eGFP+ cells in the liver (mPGK n=8; CAG n=6; hCMV n=6; CBA n=8; hSyn n=6). (C) Average ALT levels were assessed 28 days after the IM delivery of rAAV2-retro with different promoters. F (5, 39) = 1.040, P=0.4081. One-way ANOVA with Tukey's multiple comparison test. There was no statistical significance between any promoter and control (control n=9; mPGK n=5; CAG n=9; hCMV n=7; CBA n=7; hSyn n=8).

The liver, an immunologically complex organ, houses numerous phagocytic cells pivotal in immune activation 24, 25. Targeting viruses to the liver can trigger an immune response, compromising safety and limiting transgene expression efficacy 4, 26. Therefore, optimizing AAV tools with negligible or low liver targeting is critical to avoid distinct systemic immune reactions 27-29. Therefore, the liver expression of rAAV2-retro vectors containing each of the five promoters was examined. After 4 weeks post IM-delivery expression, liver imaging was performed, and fluorescence was quantified to evaluate eGFP expression levels. The results showed very low, if any, fluorescence levels in the liver (Figure 8A and 8B). Moreover, to assess the risk of hepatotoxicity, alanine aminotransferase (ALT) levels were measured. The results showed that there was no increase in ALT levels following the IM administration of rAAV2-retro vectors with any promoter (Figure 8C). This showed the absence of adverse histopathologic effects in the liver after rAAV2-retro vector expression.

The aforementioned *in vivo* experiments were all conducted on neonatal mice. Additionally, we performed parallel experiments using the rAAV2-retro vector harboring the CAG promoter to target expression in CNS of adult mice. Despite administering a high dosage of 1x10¹¹ genome copies through a single intramuscular injection, the observed expression within the CNS was markedly weak (Supplementary Figure S3). Specifically, within the brain, only a scant number of eGFP-positive cells were detected, localized to the medulla region of the brainstem. Likewise, in the spinal cord, notable eGFP expression was restricted to a few cells within the lumbar segment, directly corresponding to the injection site in the gastrocnemius muscle, whereas other segments of the spinal cord exhibited minimal or no expression at all.

## Discussion

The gene therapy efficacy is based on achieving specific and adequate therapeutic gene expression. Key variables influencing the AAV vectors' transduction efficiency include the promoter choice as well as the serotype of the viral vector. The current study aimed to characterize expression profiles driven by five promoters, including mPGK, CAG, hCMV, CBA, and hSyn to optimize rAAV2-retro-mediated LMN transduciton in the brainstem and spinal cord after the IM injection. Given the promoter size importance in accommodating complex cargo, bi-cistronic proteins, and large transgenes using AAVs 30-32, promoters with lengths not exceeding 1602 bp were selected for this study, allowing for a 3200-bp cargo. The promoters used in the current study are commonly utilized to transfer gene in the nervous system, which make them suitable for gene therapy applications. Our findings demonstrated robust transgene expression levels in the spinal cord and brainstem after the IM injection of the rAAV2-retro vector into the unilateral gastrocnemius muscle of newborn mice, with minimal expression levels observed in the liver. Notably, the CAG and hCMV promoters exhibited strong transgene expression levels in either the brainstem or spinal cord. However, mPKG failed to directly transduce the brainstem, while hSyn did not directly transduce the spinal cord. The vast majority of transduced cells driven by these promoters in the CNS were identified as LMNs. Moreover, efficient expression of the rAAV2-retro vector driven by hCMV relied on the presence of both WPRE and β-globin intron. Importantly, none of the promoters induced hepatotoxicity, highlighting their safety profiles for gene therapy applications.

The promoter, an upstream region of a gene that is crucial for initiating transcription, plays a pivotal role in gene expression regulation within gene therapy vectors. Careful selection of a promoter can enhance the specificity of a gene expression as well as its expression levels 33-35. Promoters are commonly categorized into two categories: cell-type-specific and ubiquitous. The promoters that typically drive a long-term and high expression of a gene across various cell types are known as ubiquitous. Examples include the hCMV promoter (encompassing the CMV enhancer and promoter), CBA promoter, and CAG promoter (a synthetic construct combining the CMV enhancer with the CBA promoter). The hCMV promoter is generally known for its robust activity in driving the expression of a gene. Nonetheless, numerous studies have indicated that the CMV promoter can be potentially inactivated or silenced in hepatocytes 36,37. The CAG promoter can drive high-level gene expression in mammalian cells 38-40 Recent data indicates that more than 50% of rAAV vectors utilized in clinical studies rely on three ubiquitous promoters: hCMV, CBA, and CAG promoters 41, 42. These promoters have been widely used in clinical studies targeting tissue- and organ-specific disorders to achieve high transgene expression levels. They exhibit robust ubiquitous expression in various cell types; however, their relatively larger sizes, ranging from approximately 650 bp (hCMV) to 1,600 bp (CAG), can pose challenges for rAAV gene therapy by limiting the cargo capacity of the vector. Conversely, small promoters, including the mPGK promoter (597 bp) and the hSyn promoter (448 bp), offer advantages in terms of compactness 43, 44 and tissue-specificity. The hSyn promoter does not exhibit ubiquitous activity and is neuron-specific. Therefore, to optimize rAAV-mediated expression, comparing and screening promoters for specific purposes and regions are imperative.

Our study revealed that CAG and hCMV promoters exhibited robust expression, while mPGK and hSyn promoters showed weak or negligible expression in the brainstem and spinal cord following the IM delivery of the rAAV2-retro viral vector. Interestingly, while both CAG and hCMV demonstrated robust expression *in vivo*, CAG displayed predominant expression *in vitro* compared to the other promoters. This suggests that delivery methods influence the expression efficiency of different promoters. Moreover, despite both CAG and hCMV driving strong expression in the CNS following IM delivery, the larger size of the CAG promoter compared to hCMV (1602 bp *vs.* 653 bp) potentially limited its payload capacity. Small-size promoters are ideal for incorporating large and complex cargo; therefore, hCMV might be a more promising candidate for further gene therapy research.

Furthermore, the results indicated that regardless of the promoter used, the expression was primarily restricted to the brainstem and spinal cord, with minimum expression observed in the choroid plexus. This suggested that the specificity of expression sites in the CNS after IM injection was influenced more by the delivery method and vector rather than the promoter. Moreover, it was found that the relative levels of expression driven by these promoters were consistent across various nervous systems, including the brainstem, spinal cord, and peripheral DRG. Specifically, the CAG, hCMV, and CAB promoters drove relatively strong expressions, while the mPGK and hSyn promoters resulted in poor expressions. This result underscored the significant impact of promoter selection on the gene expression level. However, the current experiments did not elucidate the high transgene expression driven by the CAG and hCMV promoters, while the mPGK and hSyn promoters-driven expression levels were significantly lower in the CNS after IM administration.

In 2016, Karpova's laboratory introduced a breakthrough by engineering strong retrograde activity into the AAV capsid through directed evolution *in vivo*, resulting in a novel variant termed rAAV2-retro 10. This advancement was speculated to be facilitated by either disrupting the binding site for hairpin mediated by insertion or creating a new binding surface incorporating the target peptide. Due to the robust retrograde expression properties and ability of sufficient sensor/effector expression for functional circuit interrogation and genome editing* in vivo* in targeted neuronal populations, rAAV2-retro-based tools have shown widespread applications not only in elucidating connectivity patterns in the CNS but also in therapeutic intervention for neurodegenerative disorders 11. Recent findings have demonstrated the broad effectiveness of rAAV2-retro in transducing LMNs in the CNS by either IM or intravenous injection in neonatal mice 12. In this study, we further quantified the efficiency of rAAV2-retro with different promoters in transducing LMNs in the spinal cord and brainstem. The results demonstrated that rAAV2-retro transduced a high proportion of the cholinergic LMNs after a single IM injection, which was independent of a specific promoter, thus suggesting a promising potential for treating MNDs.

It is worth noting that while rAAV2-retro vectors driven by different promoters induced relatively high selective expression in both the spinal cord and brainstem, and a significant proportion of the transduced cells were ChAT+ neurons, a considerable number of ChAT- cells also transduced. Additionally, some cells in the choroid plexus were observed to be transduced, further emphasizing the non-specificity of rAAV2-retro-mediated expression. Our previous findings suggest that the vectors may have disseminated throughout the system via the bloodstream or cerebrospinal fluid (CSF) 12. Specifically, we detected the presence of rAAV2-retro particles in both CSF and blood samples collected from injected mice. Furthermore, when spinal cord transection injury was induced prior to injection, the extensive expression pattern of rAAV2-retro in the spinal cord was disrupted, underscoring the importance of CSF circulation for the widespread distribution of these vectors. Despite this non-specificity, the observed expression pattern still predominantly targeted the nervous system, particularly the ChAT+ neurons, which are crucial for motor function. The rAAV2-retro vectors, with their robust retrograde expression capabilities, demonstrate potential for therapeutic applications in MNDs. However, the non-specific expression highlights the need for further optimization of vector design to enhance target specificity and minimize off-target effects.

It's crucial to explore the mechanisms behind nonspecific transgene expression in brain neurons following IM delivery of rAAV2-retro. Since there's no direct innervation from the brainstem to limb muscles 45, direct viral transduction via neuronal axons is ruled out. Beyond systemic spread via the bloodstream or CSF, could trans-synaptic transmission in neonatal mice explain the transgene expression observed in the brainstem? Unlike rabies or pseudorabies viruses, AAV vectors aren't currently known to undergo trans-synaptic retrograde transmission 46. However, the unique attributes of neonatal mice might facilitate such transmission. The potential for viral particles to cross synaptic junctions and transduce interconnected neurons (trans-synaptic transmission) deserves further investigation. Confirmation would raise questions about the timing of transmission, its persistence beyond the neonatal period, and its specificity to certain developmental stages. Additionally, exploring whether pathological conditions can trigger or enhance trans-synaptic transmission, and identifying the mechanisms involved (such as neuron shedding, blood leakage, or unique features of the neonatal brain) are essential for future research aimed at enhancing the specificity and efficacy of rAAV2-retro-mediated gene therapy.

The specificity of viral vectors in targeting different neuronal populations is crucial for enhancing therapeutic efficacy and minimizing adverse effects in gene therapy for central nervous system disorders. Various strategies, including capsid engineering, incorporation of specific elements, and selection of promoters, have been explored to improve the efficiency of viral vector expression towards particular neuronal types. For instance, Z. Lu's lab recently developed a novel retrograde AAV capsid, AAV-DJ8R, which efficiently transduced cortical projection neurons following striatal delivery in mice and macaques, successfully mediating the expression of opsins to facilitate functional manipulation of neurons 47. The C.J. Buchholz's lab enhanced cell-type specific delivery of AAV2 to interneurons by performing capsid engineering, displaying the designed ankyrin repeat protein 2K19 specific for the glutamate receptor subunit 4 at the N-terminus of the VP2 capsid protein 48. Additionally, incorporating the Neuron Restrictive Silencer Element (NRSE), which serves as a negative regulator of transcription in non-neuronal cells, into AAV-E1.1 predominantly drove expression in inhibitory neurons 49. With regards to neuronal specificity in promoter selection, beyond the extensively studied SYN promoter, a truncated human choline acetyltransferase (ChAT) promoter has been validated to facilitate efficient and selective expression of cholinergic neurons 50. Our current study just compares several commonly used promoters. It is imperative to further explore the potential of other promoters, such as the ChAT promoter, to potentially enhance the specificity of rAAV2-retro infection towards motor neurons in the spinal cord.

AAV has numerous features; however, its packaging capacity is limited to about 4,700 bp, which is roughly half the capacity of lentiviral or adenoviral vectors. Increasing the payload ability of AAV vectors is a major challenge for effectively delivering therapeutic genes. For this purpose, we investigated whether we could abandon some elements to reduce the backbone size of the vector and consequently increase its cargo capacity. The impact of WPRE on gene expression varies depending on the promoter used. For instance, its effectiveness on retroviral vectors differs when paired with a heterologous mouse mammary tumor virus (MMTV) promoter or whey acidic protein (WAP) promoter. Interestingly, the beneficial effects of WPRE might not hold for all promoters; in some cases, its combination with the WAP promoter resulted in decreased gene expression levels in certain cell lines 22. These results indicated that the effects of WPRE on gene expression are very complex. Therefore, when incorporating WPRE elements into gene therapy vectors, it is essential to consider the characteristics of the specific promoter used, as this can significantly influence overall transgene expression efficacy. The current study investigated the necessity of WPRE for expression driven by CMV promoter and evaluated the feasibility of abandoning WPRE to maximize the cargo space of rAAV2-retro vectors carrying the CMV-driven transgenes. The impact of abandoning β-globin, a regulatory element commonly utilized as a gene expression enhancer and located in or near the gene, was investigated for a similar purpose. However, our results revealed that the absence of either WPRE or β-globin significantly reduced the expression driven by hCMV in the spinal cord, indicating the necessity of both elements for achieving efficient expression directed by hCMV.

This work demonstrates a comparison of the expression efficiency of rAAV2-retro in the central nervous system of neonatal mice driven by different commonly used promoters. While rAAV2-retro demonstrates limited expression efficiency in adult mice, it exhibits robust expression in neonatal mice, with particularly high specificity towards ChAT+ neurons in the spinal cord, which holds particular importance in the context of genetic motor neuron diseases, such as SMA and certain cases of ALS. Genetic newborn screening for motor neuron diseases, coupled with early therapy, may significantly enhance treatment outcomes 51. However, improving the expression efficiency of rAAV2-retro in adult animals remains a critical prerequisite for broadening its therapeutic applications.

## Conclusions

This comparative study of rAAV2-retro promoters provided valuable insights for the streamlined, safe, and effective development of future AAV-based gene therapy treatments for neuromuscular disorders, such as MNDs. The result showed that combining the hCMV promoter with the rAAV2-retro viral vector serotype might emerge as the optimal choice for delivering transgenes via IM injection to the LMNs within the CNS of neonatal mice.

## Supplementary Material

Supplementary figures.

## Abbreviations

AAV: adeno-associated viral vector
IM: intramuscular
LMN: lower motor neuron
CNS: central neural system
mPGK: mouse phosphoglycerate kinase
CAG: CMV early enhancer/chicken β-actin/short β-globulin intron
hCMV: human cytomegalovirus
CBA: chicken β-actin
hSyn: human synapsin
WPRE: woodchuck hepatitis post-transcriptional regulatory element
