# Supplementary material for: Intramuscular Injection of rAAV2-retro for Low Motor Neuron Transduction: Evaluating Five Promoters
Source: Int J Med Sci. 2025 Jan 21;22(4):775–89. doi: 10.7150/ijms.101807 (PMC11843134; doi:10.7150/ijms.101807)
Supplement: Supplementary file 1 — Supplementary figures. [file ijmsv22p0775s1.pdf]

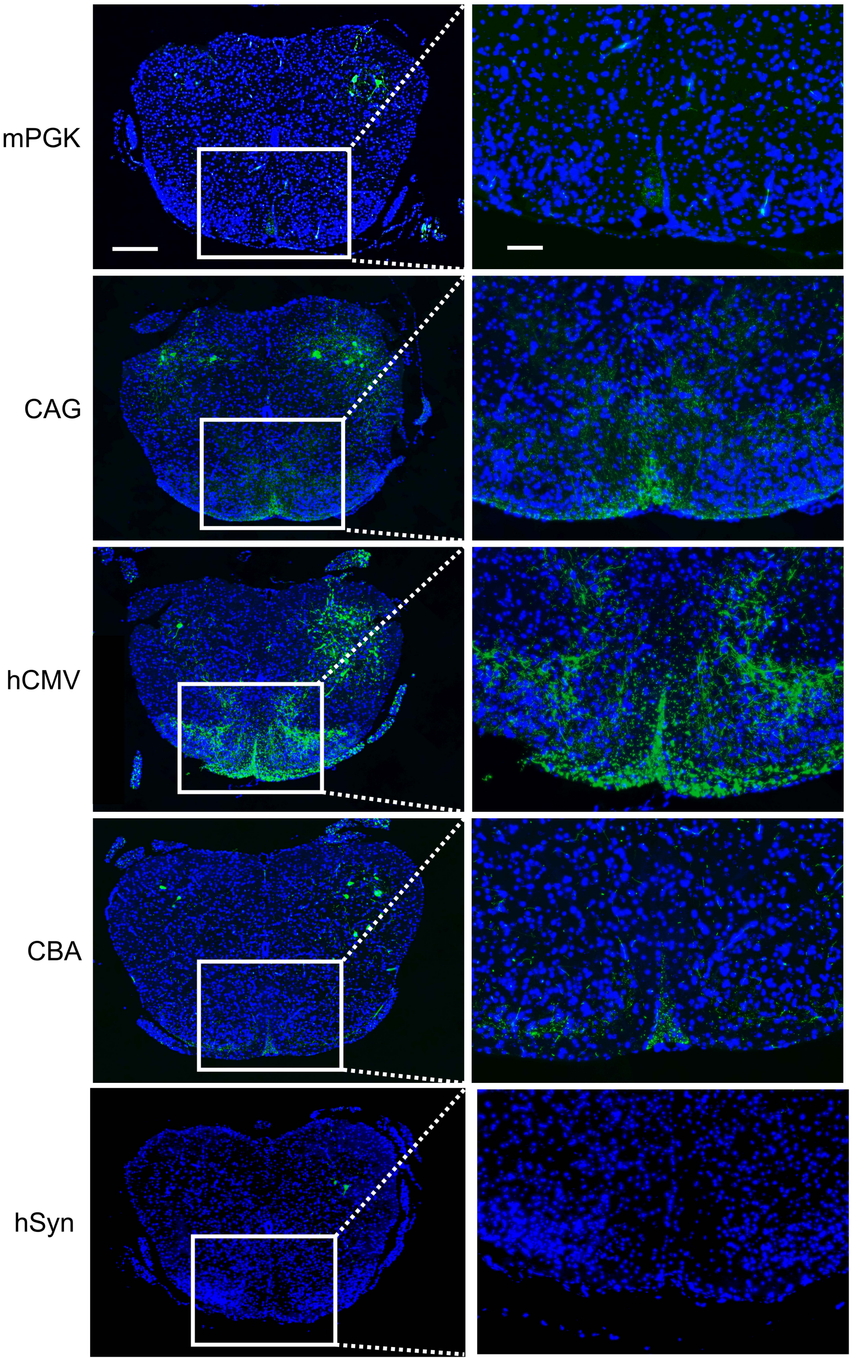

Supplementary Figure S1. eGFP expression driven by different promoters in the posterior horn of spinal cord transfected with rAAV2-retro vectors. Left scale bar, 250um; Right scale bar, 100um.

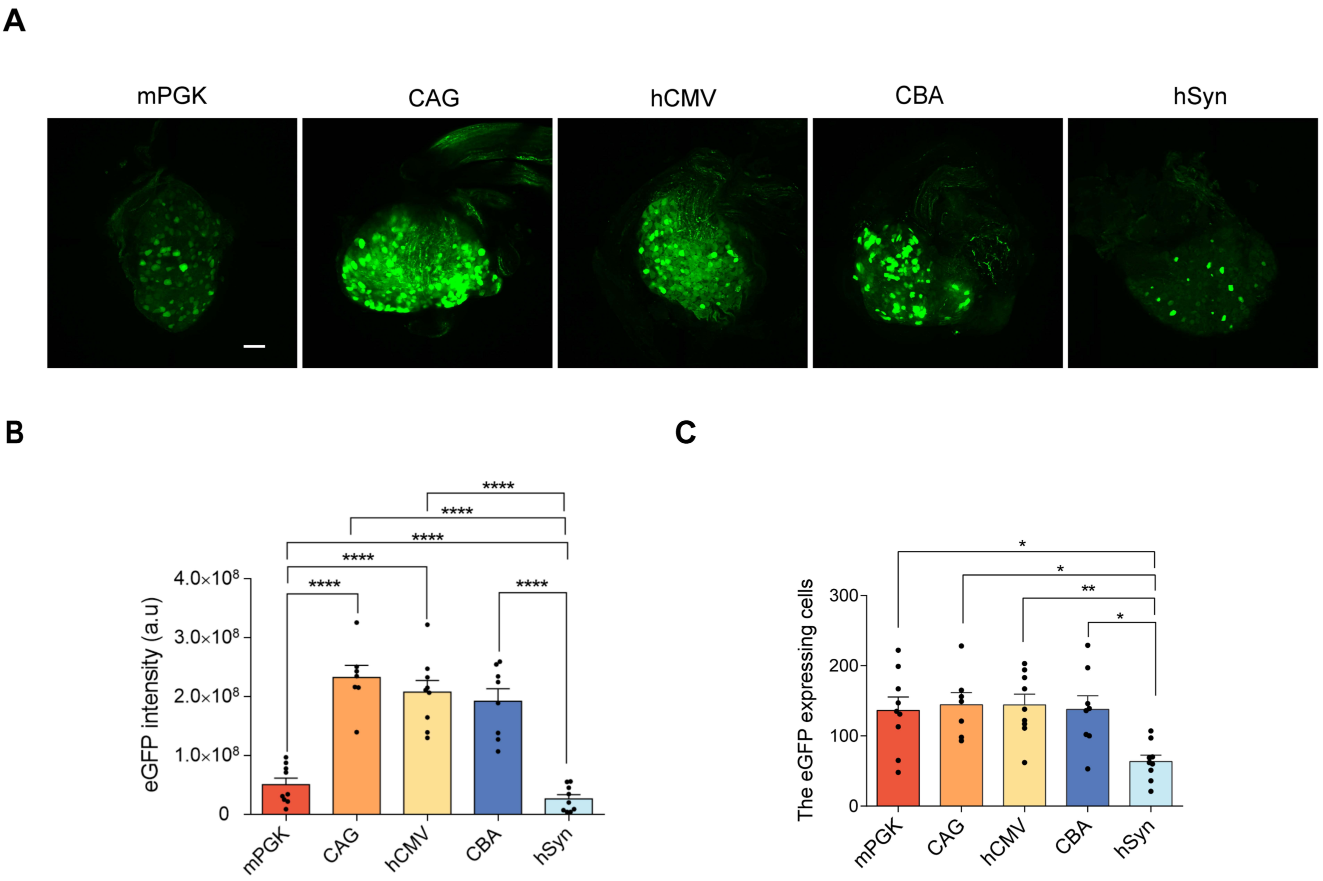

Supplementary Figure S2. Intramuscular injection of rAAV2-retro resulted in expression of DRG neurons. (A) Representative maximum intensity projections (MIPs) of z stacks from 3D visualization of DRG (scale bar, 200 µm). (B) Mean eGFP intensity quantification in DRG. F (4, 37) = 34.07, P < 0.0001. One-way ANOVA with Tukey's multiple comparison test. (C) Total number of native eGFP-expressing cells in the DRG. The DRGs examined were from the L1 segment of the spinal cord, ipsilateral to the injection site. F(4, 37) = 4.676, P = 0.0037. Data presented as mean±SEM (mPGK n=9; CAG n=7; hCMV n=9; CBA n=8; hSyn n=9). *P <0.05, **P <0.01, ****P <0.0001, one-way ANOVA with Tukey's multiple comparison test.

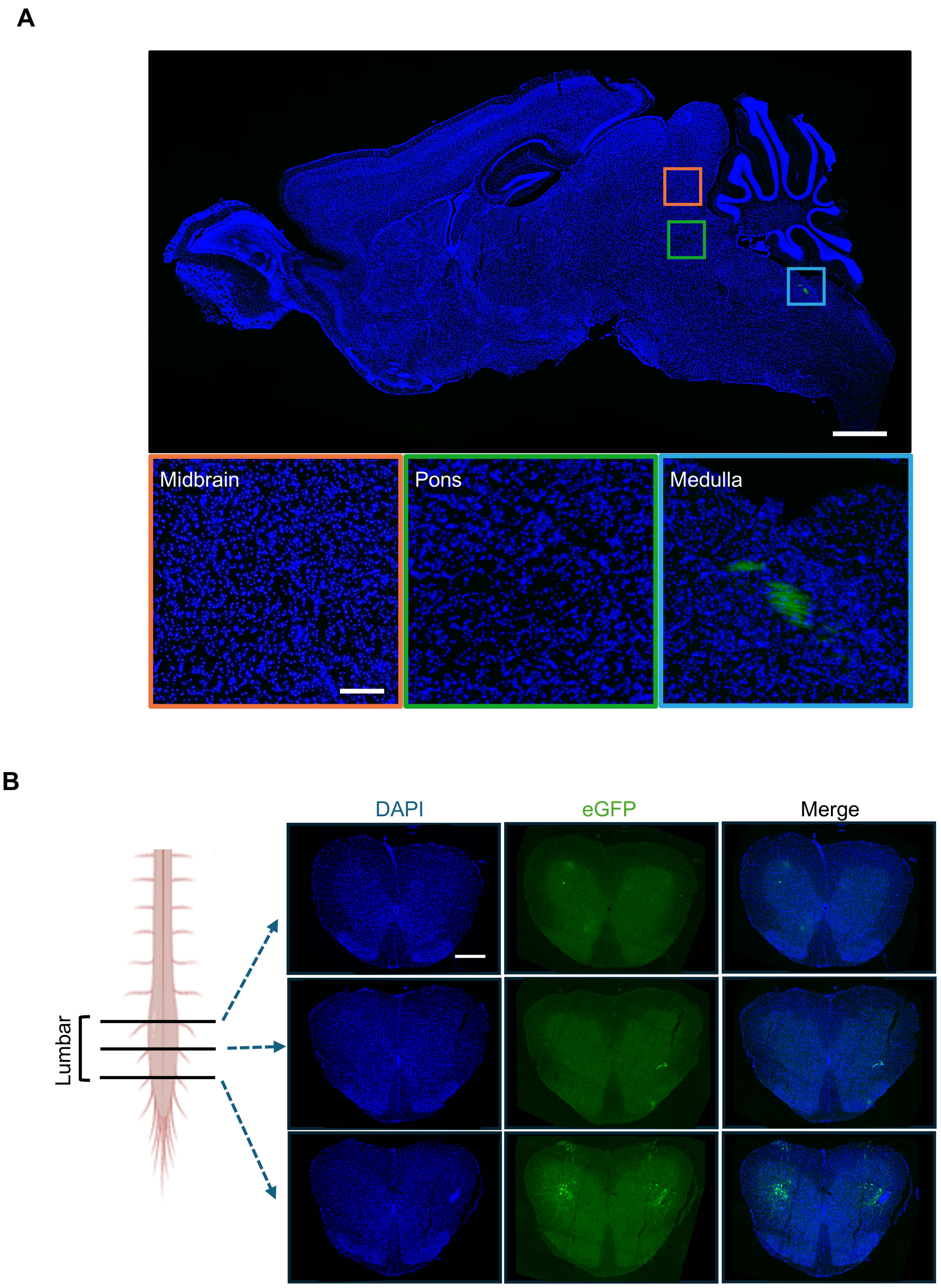

Supplementary Figure S3. Labeling in the central nervous system 4 weeks after gastrocnemius single injection of rAAV2-retro-CAG-eGFP (1X10^{11} genome copies) in adult mice. A. Labeling in the brain. The upper panel depicts an overall sagittal view of the brain (scale bar, 1000µm) The lower panel presents magnified views of the subregions within the brainstem (scale bar, 100µm) B. Labeling in lumbar segment of spinal cord (scale bar, 400µm).
